# Acute effects of a commercially-available pre-workout supplement on markers of training: a double-blind study

**DOI:** 10.1186/s12970-014-0040-0

**Published:** 2014-08-15

**Authors:** Jordan J Outlaw, Colin D Wilborn, Abbie E Smith-Ryan, Sara E Hayward, Stacie L Urbina, Lem W Taylor, Cliffa A Foster

**Affiliations:** 1 Human Performance Lab, University of Mary Hardin-Baylor, 900 College Street, Belton 76513, TX, USA; 2 Department of Exercise and Sport Science, University of North Carolina Chapel Hill, 209 Fetzer Hall, CB# 8700, Chapel Hill 27599, NC, USA

**Keywords:** Body composition, Pre-workout, Creatine, Beta-alanine, Strength

## Abstract

**Background:**

Pre-workout supplements containing numerous ingredients claim to increase performance and strength. Product-specific research is important for identifying efficacy of combined ingredients. The purpose of this study was to evaluate the effects of a proprietary pre-workout dietary supplement containing creatine monohydrate, beta-alanine, L-Tarurine, L-Leucine, and caffeine, on anaerobic power, muscular strength, body composition, and mood states.

**Methods:**

In a double-blind, randomized, matched-pair design, twenty male subjects (mean ± SD; 22.4 ± 9.5 yrs, 76.9 ± 11.2 kg, 22.7 ± 9.5% body fat), consumed either 30 g of a pre-workout supplement (SUP) or maltodextrin placebo (PLC) 30 minutes before a resistance training workout, after completing baseline testing. Body composition was determined via dual-energy x-ray absorptiometry (DEXA). Subjects completed 12 vertical jumps for height (VJ) and one repetition maximum (1RM) and repetitions to failure lifts on bench (BPM) and leg press (LPM). Finally, subjects completed a Wingate power test on a cycle ergometer [mean power (WMP) and peak power (WPP)]. After baseline testing, participants completed eight days of supplementation and four split-body resistance-training bouts. Side effect questionnaires were completed daily 30 minutes after consuming the supplement. Subjects completed post-supplement testing on Day 8. Data were analyzed utilizing a 2 × 2 repeated measures ANOVA [treatment (PLC vs SUP) × time (T1 vs T2)] and ninety-five percent confidence intervals.

**Results:**

There were no significant treatment × time interactions (p > 0.05). There were no significant changes in %body fat (%BF; ∆-0.43 ± 0.58; p = 0.920), fat mass (∆-2.45 ± 5.72; p = 0.988), or lean body mass (LBM; 10.9 ± 12.2; p = 0.848). 95% CI demonstrated significant LBM increases for both groups. There was a main effect for time for WPP (∆100.5 ± 42.7W; p = 0.001), BPM (∆8.0 ± 12.9 lbs; p = 0.001), and LPM (∆80.0 ± 28.8 lbs; p = 0.001), with no significant differences between treatments. There was no significant difference in mood states between groups or over time.

**Conclusion:**

The proprietary pre-workout blend combined with eight days of training did not significantly (ANOVA) improve body composition or performance. While not significant, greater gains in LPM were demonstrated in the SUP group for lean body mass and lower body strength. Future studies should evaluate more chronic effects of proprietary pre-workout blends on total training volume and performance outcomes.

## Background

Scientific research on ergogenic supplements has led manufacturers to introduce pre-workout drinks to the market. Supplements taken before a workout are often used to improve energy, alertness, strength, power, and body composition. To date, little product-specific research exists on pre-workout supplements containing multiple ingredients. Consumption of various individual ingredients often included in proprietary pre-workout blends are known to effectively increase time to fatigue [1],[2], allowing for an increased training volume [3]-[5] although it is uncertain which combinations of ingredients are most effective. The following scientific support serves as the basis for formulation of proprietary blends and the inclusion of specific ingredients.

Beta-alanine, a precursor to carnosine [6], has been used to improve performance of high-intensity exercise [7],[8] by increasing the muscle carnosine pool [9]. Carnosine serves as a muscle buffer during intense exercise and increasing carnosine stores through beta-alanine supplementation can enhance this buffering ability [6]. Research on beta-alanine has shown that supplementation improves the rate of fatigue in sprinters [10] and improves YoYo Intermittent Recovery performance (the ability to repeatedly perform and recover from exercise) for amateur athletes [7]. Additionally, beta-alanine supplementation has increased the number of repetitions to fatigue and overall work capacity [6].

Creatine, the most extensively researched ergogenic aid [11], has been shown to increase strength and improve body composition in most individuals when combined with exercise [12]. Creatine’s ergogenic abilities are derivative of its ability to rapidly replenish ATP stores, allowing for quicker recovery and potential increased training volume [13]. To properly load creatine stores in the muscle, it is recommended that an individual consume roughly 0.3g/kg/day for three days followed by a maintenance dose of 3-5g/day after the first three days [11]. Alternately, a lower dose of 2-3g/day may also be utilized to increase stores slowly [11]. Supplementation of creatine is also beneficial for improving lean body mass when combined with exercise [14]. According to the International Society of Sport Nutrition Position Stand on Creatine, creatine monohydrate is currently the most effective supplement for increasing anaerobic capacity and lean body mass [11].

Research on branched chain amino acids (BCAA) has concluded that supplementation can result in increased protein synthesis and additional lean body mass in multiple populations [6]. BCAA have also been shown to improve muscular strength as well as an increase thigh mass [15]. Muscle damage has been markedly reduced after exercise and BCAA supplementation [16]. Intake of BCAA, and leucine in particular, can create an anabolic environment [17] by reducing protein oxidation and promoting sarcomerogenesis in skeletal muscle [18]. The inclusion of BCAA before or after an exercise session will help the body maintain a positive nitrogen balance and support muscle growth.

Another well researched ergogenic aid, caffeine, is often a key component to pre-workout supplements due to its stimulatory benefits, and subsequent improvements in time to fatigue [6]. Caffeine has also been shown to have a potential glycogen-sparring effect during exercise, likely improving endurance [19], and chronic beneficial changes in body composition [20]-[22]. A dose of 3-6mg/kg of body weight can improve sport performance in trained individuals [19]. Multiple studies have resulted in increased upper body strength [23],[24] while still others have not seen the same results [25],[26]. Based on varying results, it appears that more research is needed to determine caffeine’s effectiveness in the area of strength and power performance.

Caffeine is also a thermogenic, which explains its inclusion in weight loss supplements [19].

Although beta-alanine, creatine, BCAAs, and caffeine are frequently the active ingredients in pre-workout supplements, different amounts can be used depending on the specific goals of the target population. Additionally, the actual degree of success and time frame for effects of multi-ingredient combinations differ for every individual and some consumers are considered non-responders [27]-[29]. The variances among formulation, composition, and timing of response can cause varying results. The purpose of this study was to determine the acute (one week) effects of a commercially available pre-workout supplement containing a proprietary blend of caffeine, creatine, BCAAs, and beta-alanine on strength, power, body composition, mood states, and tolerance measures when combined with a selected resistance four day training protocol.

## Methods

### Participants

Twenty males (mean ± SD; 22.4 ± 9.5 years, 76.9 ± 11.2 kg, 22.7 ± 9.5% body fat) volunteered for the study. Participants were recruited for inclusion if they were healthy, resistance-trained (participated in a structured resistance training protocol for the past 36 months) males, able to bench press 120% of their body weight and leg press 2.5 times their body weight. The study protocol and procedures were approved by the University IRB committee prior to the start of the recruitment process and participants completed medical and exercise history surveys, as well as signed the written Informed Consent prior to study initiation. Participants were screened for inclusion/exclusion criteria by laboratory assistants. Volunteers were excluded from the study if they had any known metabolic disorders, history of pulmonary disease, hypertension, liver or kidney disease, musculoskeletal or neuromuscular disease, neurological disease, autoimmune disease, or any cancers, peptic ulcers, or anemia. Exclusionary measures also included having taken ergogenic levels of nutritional supplements that may affect muscle mass or aerobic capacity (e.g., creatine, beta-hydroxy-beta-methylbutyrate) or anabolic/catabolic hormone levels (e.g., androstenedione, dehydroepiandrosterone, etc.) within six months prior to the start of the study.

### Procedures

#### Baseline testing

Participants completed all testing measures in the following order: dual-energy x-ray absorptiometry (DEXA), vertical jump (VJ), bench press one repetition maximum (BPM), bench press repetitions to failure (BPRep; as many repetitions as possible at 85% BPM), leg press one repetition maximum (LPM), leg press repetitions to failure (LPRep; as many repetitions as possible at 85% LPM), and Wingate. Baseline measurements were determined on Day 0 (T1) before beginning the supplementation and training protocol. Participants completed a 4-day baseline diet log prior to testing, reporting all dietary intake (food, method of preparation, and quantity). All subjects were required to refrain from exercise for the 24-hours prior to testing.

#### Body composition

A DEXA scan (Discovery QDR, Hologic, Inc., Bedford, MA) was utilized to measure body composition. Participants were positioned on their back and required to remain still for the six-minute scan. Body fat percentage (%BF), fat mass (FM) in grams, and lean body mass (LBM) in grams were determined by and recorded from the DEXA scan report.

#### Vertical jump

A measure of power output [30], Vertical Jump (VJ) was determined using the Vertec Jump Trainer (Sport Imports, Columbus, Oh.) following guidelines established by the National Strength and Conditioning Association (NSCA) [31]. While following standard VJ procedures, each subject was allowed 12 attempts to reach their peak height. Jump measurements for all 12 attempts were recorded by a trained lab assistant in inches. Participants rested for one minute after each jump attempt. Participants were given 12 attempts to reach a true vertical jump height as pre-testing indicated that participants were still increasing jump height after 8–10 jumps.

#### Strength measures

Participants completed 2 sets of 8–10 repetitions of bench press on the dynamic Hammer Strength bench press (Life Fitness, Rosemont, IL.) at approximately 50% of anticipated max to prepare for the upper body strength tests. Participants then performed successive lifts starting at roughly 70% of anticipated 1 repetition maximum (1RM) and increasing by 5 – 10 lbs after each successful lift until reaching a 1RM. Bench press maximum was recorded as the most weight they were able to lift before failure or a lift requiring assistance. A one repetition maximum on bench press was reached within three lifts on average. Participants were allowed to perform the lift at a self-selected pace, as long as the bar was lowered to the chest and pressed upward until the elbows were fully extended. After resting for five minutes, participants completed maximal repetitions at 85% of established BPM for a repetitions to failure measure (BPRep). Participants were instructed to complete as many repetitions as possible while maintaining required points of contact, touching their chest (without bounce) with the bar before returning to the start position, and without resting between each lift. A lab assistant counted repetitions until the participant could no longer maintain a steady rhythm or was unable to perform the exercise, at which point the participant was instructed to cease lifting.

A warm-up on the plate-loaded leg press (Life Fitness, Rosemont, IL.) (2 sets of 8 – 10 repetitions at approximately 50% of anticipated maximum) was completed before subjects attempted 1RM lifts. Starting at about 70% of anticipated 1RM, weight was increased by 10–25 lbs until participants reached a leg press 1RM. Participants were required to keep both feet flat on the foot platform and to lower the weight until their knees were at a 90° angle (signaled by a lab assistant), as per standard procedures. Once reaching the 90° mark, participants returned to the starting position without the help of lab personnel or pushing on their legs with their hands. On average, participants reached their 1RM within three attempts. Subjects again rested for five minutes then completed maximal repetitions at 85% of 1RM for a measure of lower extremity muscular repetitions to failure (LPRep). Lab assistants counted repetitions and instructed the participant on when to stop the exercise in a similar fashion as was done with BPRep.

#### Wingate anaerobic power test

Following a 15 minute rest, subjects performed the Wingate Load Test on the LODE Excalibur Sport Ergometer (Lode BV, Groningen, Netherlands). The Wingate testing protocol consists of a two minute warm-up period in which the participant was instructed to maintain a cadence of 60–80 rpm followed by a 30 second sprint. When five seconds remained in the warm-up period, the participant started pedaling as hard and as fast as they could for the next 30 seconds while remaining seated. Over the course of the 30 second testing period, the resistance placed on the fly wheel remained constant at 0.75 N∙m∙kg^−1^[32]. Peak power in Watts (WPP) and Watts produced over the course of the test and averaged to create a mean power (WMP) measure were recorded.

#### Diet log

Participants turned in a nine day diet log to lab personnel containing all foods and beverages consumed (type, brand, method of preparation, amount consumed) for the duration of the study (Day 0-Day 8). Diet logs were analyzed by lab personnel utilizing The Food Processor (esha Research, Salem, OR) Nutrition and Fitness Software.

#### Supplementation and training

The study was conducted in a double-blind, placebo controlled manner with participants consuming either 30 g of the placebo (PLC; n = 10; maltodextrin) or 30 g of the active supplement (SUP; n = 10; XPAND2X®, Dymatize Enterprises, LLC., Dallas, TX) dissolved in eight to ten fluid ounces of water for eight days. The placebo and supplement were matched for color and taste and both were in powder form. All participants were matched according to their T1 LBM (SUP: 62525.21 ± 8023.39 g; PLC: 64753.17 ± 7026.71 g) and randomly assigned to either PLC or SUP. The active supplement contained 8.4 g of creatine monohydrate-beta-alanine blend, 4.8 g of BCAAs, and 275 mg of total caffeine in the 30 g serving. Thirty grams (three scoops) was the suggested serving size for experienced users per the manufacturer.

All subjects reported to the Human Performance Lab and completed the resistance training protocol under lab assistant supervision on four different days (Monday, Tuesday, Thursday, and Friday) within one week. Because the purpose of this study was to examine the acute effects of the supplement combined with resistance training, four workout sessions within the testing week appeared to be the most effective number of training sessions for the given amount of time. Thirty minutes prior to their workout, participants were asked to come to the Human Performance Lab to consume their assigned pre-workout beverage. To allow for proper nutrient absorption after intake, participants were required to wait 30 minutes before beginning their workout. During the 30 minute waiting period, participants remained in the Human Performance Lab.

Participants completed four resistance-training, split-body workouts consisting of 10 exercises, each performed for 3 sets of 8 repetitions with as much weight as was tolerated to lift per set (targeting 80% of 1RM) and one core exercise with 20 reps for 3 sets (Table 1). The participant rested for 1 minute between sets and for 2 minutes between exercises. Workouts were monitored by a trained research assistant to ensure the quality of each workout. Three hours following completion of each training session, participants completed a side-effects questionnaire to monitor and assess tolerance associated with pre-workout supplementation. On non-workout days, participants consumed their assigned supplement during the morning hours and completed the side effects questionnaire three hours post-consumption.

**Table 1:** Training protocol

	**Workout A**	
**Exercise**	**Sets**	**Reps**
Squat	3	8
Leg Extension	3	8
Seated Calf Raises	3	8
Hamstring Curls	3	8
Dumbbell Incline Press	3	8
One-arm Dumbbell Rows	3	8
Shoulder Press	3	8
Dumbbell Curls	3	8
Triceps Pushdowns	3	8
Deadlifts	3	8
Crunches	3	20
	**Workout B**	
**Exercise**	**Sets**	**Reps**
Leg Press	3	8
Lunges	3	8
Standing Calf Raises	3	8
Deadlifts	3	8
Bench Press	3	8
Seated Rows	3	8
Lat Pulldowns	3	8
Side Laterals	3	8
Barbell Curls	3	8
French Press	3	8
Russian Twists	3	8

Two split-body workouts were designed and utilized. Workout A was completed on Day 1 and Day 4. Workout B was completed on Day 2 and 5. All exercises (except crunches and Russian twists) were performed at roughly 80% max intensity. Participants recorded weight used and number of repetitions achieved for each exercise.

#### Post-supplementation testing

On Day 8, after seven days of supplementation, all of the testing parameters (DEXA, HR, BP, VJ, BPM, BPRep, LPM, LPRep, Wingate) were repeated (T2). Participants rested the day before T2 and again completed and turned in a four-day diet log. Thirty minutes before final performance testing, participants consumed their pre-workout drink for the 8^th^ and final time. The side-effects survey was completed three hours post T2. Participants reached their 1RM for both bench press and leg press within three lifts on average.

### Data analysis

Separate two-way repeated measures ANOVAs [treatment (SUP vs PLC) × time (T1 vs T2)] were used to analyze %BF, FM, LBM, body mass, HR, BP, VJ (peak), BPM, BPRep, LPM, LPRep, WPP, and WMP. When significance was found, Bonferroni-corrected post-hoc comparisons were used. An alpha level was set at 0.05, and all data were analyzed using SPSS (Version 20.0 Chicago, IL, USA). Ninety-five percent confidence intervals were constructed around the mean change scores. When the 95% confidence interval included zero, the score was not deemed statistically significant. A Kruskal-Wallace one-way analysis of variance was used to interpret all survey data.

## Results

**Figure 1:**
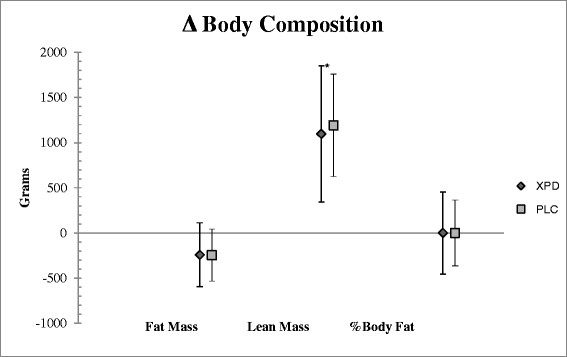
**Body composition measures.** Change in body composition measures from baseline values. Lean Mass was significantly increased for PLC and SUP from baseline to final testing. There were no significant changes for Fat Mass. *indicates a significant time effect (p ≥ 0.05).

There were no significant group x time interactions (p > 0.05) for body composition, LPM, BPM, WPP, WMP, or VJ, and no effects for treatment. There was a significant effect for time for FM (p = 0.05; η_p_^2^ = 0.196), LBM (p = 0.001; η_p_^2^ = 0.551), and %BF (p = 0.008, η_p_^2^ = 0.335). Mean difference values (±95% CI) depict the significant increase in LBM for both groups (Figure 1).

**Figure 2:**
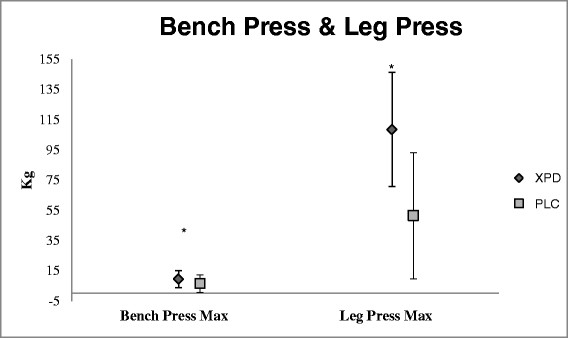
**Bench press and leg press 1RM.** Changes in BPMax and LPMax were significant for both groups from baseline testing to final testing. There was no group x time interaction. *indicates significant changes from baseline (p ≥ 0.05).

**Figure 3:**
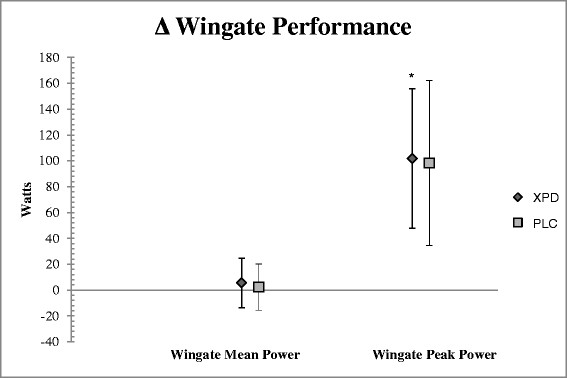
**Wingate measures of power.** Changes in WMP were not significantly different from baseline testing. WPP changes were significant for both PLC and SUP from baseline to T2 testing. There was no group x time interaction. *indicates significant changes over time (p ≥ 0.05).

There was a significant time effect for WPP (p = 0.001; η_p_^2^ = 0.550), BPM (p = 0.001; η_p_^2^ = 0.448), and LPM (p = 0.001; η_p_^2^ = 0.632); with no group x time effect for VJ (p = 0.451), or WMP (p = 0.563). Mean difference scores (±95% CI) depict significant increases in BPM, LPM, and WPP, with no differences between groups (Figures 2 and 3). However, SUP group had an increase in leg press max that was two times greater than that of the PLC group. There was no significant difference between groups for total calories (*p* = 0.296), grams of fat (*p* = 0.880), grams of protein (*p* = 0.884), or grams of carbohydrate consumed (*p* = 0.170). See Table 2 for nutritional intake data. The most often reported side-effects after supplementation were feeling faint, feeling light-headed, dizziness, headache, and nausea. These side-effects were reported by participants in both groups and therefore may or may not be attributable to the supplement.

**Table 2:** Macronutrient and caloric intake by group

	**SUP**	**PLC**
Total Calories	2320.71 ± 664.44	2352.75 ± 570.37
CHO (grams)	259.92 ± 87.25	271.90 ± 66.58
Fat (grams)	91.02 ± 30.01	99.95 ± 40.39
Protein (grams)	105.78 ± 28.45	108.05 ± 31.42

Macronutrient and Calorie information presented as mean ± SD. Food intake was recorded daily throughout the study. There was no significant difference between groups in nutritional intake.

## Discussion

Acute ingestion of a pre-workout supplement blend demonstrated positive effects on post-supplementation/training LPM, more than a carbohydrate PLC, when combined with four resistance training sessions. The improvements in LPM (+14.09 ± 6.94 kg) from T1 to T2 for SUP (PLC: LPM: +5.48 ± 7.93 kg) were likely due to a delayed onset of fatigue, attributable to the beta-alanine and creatine content of the supplement. Hoffman and associates [33] discussed the combined effects of creatine and beta-alanine supplementation on delayed fatigue and ultimately increased training stimulus. Although four training sessions may be seen as an insufficient amount of time to significantly increase strength, the supplementation of these two ingredients, combined with a hypertrophy-focused resistance training protocol, may have allowed for increases in lower body strength in the present study. This is supported by Derave et al. [10] in which dynamic knee extension torque was significantly improved after four weeks of 4.8 g/day of beta-alanine supplementation. The improvements in strength in the study by Derave et al. as well as the present findings could be linked to a potential increase in training volume often allowed by increased beta-alanine and carnosine in the body [34]. Hoffman and colleagues also saw a trend toward significance for Wingate anaerobic power tests (*p* = 0.07) with three weeks of supplementation.

The absence of a supplement loading period may have negatively impacted the study. Beta-alanine does require a loading period and while creatine does not, it is often recommended that users follow a specific protocol for the first few days of supplementing with creatine to decrease the time to results [11],[35]. Because the supplementation period was only eight days in duration, a loading period would likely have been beneficial for the SUP group. Buford and colleagues [11], in a review, suggest that creatine benefits will likely occur without a loading period, but may take four or more weeks to happen. In a study investigating different levels of dosing for beta-alanine in untrained males, a higher dose (3.2 g beta-alanine/day for four weeks, followed by 1.6 g beta-alanine/day for four weeks) more quickly increased muscle carnosine levels compared to the low dose (1.6 g beta-alanine/day for eight weeks), providing supporting evidence that beta-alanine may be more beneficial in a more timely manner when received in higher doses initially (loading) [35].

Caffeine contained in the supplement may have contributed to the increase in lower body strength, although this would be a contradictory finding as much of the caffeine research resulted in no significant lower body strength increase [19],[23],[36]. Supplementing with caffeine before a workout has been shown to increase the amount of weight lifted during the chest press exercise although not the leg press [24]. Lower body testing was again not affected by caffeine consumption in a 2006 study by Beck and colleagues [37]. Bench press 1RM was significantly increased after caffeine ingestion, but lower body strength and power (Wingate) were not changed. Although caffeine may have ergogenic effects on upper body strength and during activities more aerobic in nature, it is unlikely that the caffeine content of the active supplement in the current study had any effect on the LPM variable. Despite this finding, caffeine likely played a role in the improvement of %BF. Supplemental caffeine is often used to increase lipolysis during exercise [38] and spare glycogen [39], a benefit that could potentially be seen if the supplement used in the present study was taken for a longer period of time. In one study, overweight participants consumed a dietary supplement containing 240 mg/day of caffeine for eight weeks and achieved a significant (p < 0.006) amount of weight loss and fat mass loss in addition to a decrease in hip girth measurements [40].

It is also plausible that the increased LPM was due to the actual combination of ingredients rather than one single ingredient in particular. A similar pre-workout supplement, when ingested for a period of three weeks, significantly increased leg press strength in recreationally-trained males [41]. The particular multi-ingredient supplement used in Spradley and associates’ research contained 300mg of caffeine as well as beta-alanine, creatine, and BCAAs included in the supplement [41]. Multi-ingredient pre-workout drinks containing a combination of caffeine, creatine, amino acids, and beta-alanine, commonly demonstrating a delay in fatigue and improved peak and mean power measures after acute supplementation [42]-[44]. One such supplemental drink was consumed by 15 trained males before each workout for eight weeks and results revealed significant improvements in strength for the experimental group [44]. This study conducted by Kudrna and colleagues demonstrates the possibility for improvements through pre-exercise supplement drinks with an adequate training and supplementation period [44]. Increased training volume (attributable to delayed onset of fatigue) was seen after trained individuals consumed 18g of a multi-ingredient ergogenic supplement drink before high intensity interval training (HIIT) sessions for three weeks [4] Ingredients in the active supplement were similar to those in the current study (BCAAs, caffeine, creatine) and although group by time interactions were not significant in Smith’s study, 95% confidence intervals suggested that the supplement was beneficial on measures of aerobic performance [4]. Considering the short duration of supplementation, comparable conclusions can be drawn, suggesting potential training benefits related to the supplement if doses of ingredients and supplementation duration are adequate.

Based on the current body of knowledge and the results of this study, the ingredients in the experimental supplement and hypertrophy-focus training regimen may have the potential to increase lower body strength in resistance-trained young males. It is likely that the participants in the SUP group would have seen a significant ergogenic benefit (improved LPM) related to the supplement and training protocol after an extended supplementation period. Data from another study investigated performance variables as well as body composition effects of the same commercially available product used in the current study but with an eight week supplementation period [14]. Results support the conclusions and findings of the present study (improved strength and anaerobic power), suggesting long-term use may have greater benefits. The time delay in measurable results between these two studies reiterates the need for analyses of longer duration on pre-workout supplements as well as acute studies to determine how quickly supplement benefits can be realized.

The lack of a crossover design is one limitation to this study. Future acute research may investigate the effects of the proprietary supplement in a crossover manner to gain further knowledge of the potential for improved performance and/or body composition. A crossover study using the supplement used in the present study would also provide higher quality side-effect information.

## Conclusions

It may be beneficial for resistance trained males to consume a proprietary pre-workout supplement containing beta-alanine, creatine, BCAAs, and caffeine when wanting to improve lower body strength. It seems likely, based on the available research, that taking the pre-workout supplement for an extended period of time in combination with exercise is safe and can lead to beneficial changes in strength and body composition.

## Abbreviations

SUP: Pre-workout supplement

PLC: Placebo

DEXA: Dual-energy x-ray absorptiometry

1RM: 1 repetition maximum

BPM: Bench press maximum

LPM: Leg press maximum

BPRep: Bench press maximal repetitions

LPRep: Leg press maximal repetitions

WMP: Wingate mean power

WPP: Wingate peak power

%BF: Percent body fat

FM: Fat mass

LBM: Lean body mass

BCAA: Branched-chain amino acids

BP: Blood pressure

HR: Heart rate

BPM: Beats per minute

VJ: Vertical jump

## Competing interests

The study was funded by Dymatize Inc. The authors do not have any competing interests.

## Authors’ contribution

JO, CW, AS, and SH prepared the manuscript. SH, SU, and JO performed data collection. SH and AS performed statistical analysis. CW was the primary investigator and CF provided administrative oversight. LM assisted with manuscript editing and revisions. All authors read and approved the final manuscript.
